# The Effect of Immunosuppressive Drugs on MMPs Activity in The Walls of Blood Vessels - A Systematic Review

**DOI:** 10.7150/ijms.54423

**Published:** 2021-01-30

**Authors:** Anna Surówka, Aleksandra Wilk, Kamila Szumilas, Karolina Kędzierska-Kapuza

**Affiliations:** 1 Department of Nephrology, Transplantology and Internal Medicine, Pomeranian Medical University, 70-111 Szczecin, Poland.; 2 Department of Histology and Embryology, Pomeranian Medical University, 70-111 Szczecin, Poland.; 3 Department of Physiology, Pomeranian Medical University, 70-111 Szczecin, Poland.; 4 Clinical Department of Gastroenterological Surgery and Transplantation, Central Clinical Hospital of the MSWiA in Warsaw, Poland.

**Keywords:** immunosuppressive drugs, metalloproteinases, inhibitors, extracellular matrix.

## Abstract

The current study focuses on the role of MMPs in the pathogenesis of the vascular damage and at the same time it offers the review referring to the influence of the immunosuppressive treatment on this interdependence. Contemporary immunosuppressive treatment constitutes of four groups of medications, such as: calcineurin inhibitors including cyclosporine A and tacrolimus; inhibitors of the inosine monophosphate dehydrogenase - the only agent from this group currently used in transplantation is mycophenalate mofetil (MMF); mTOR inhibitors, consisting of everolimus and glucocorticosteroids. Due to the fact that the properties of immunosuppressive drugs still remain unclear and transplant recipients need to use these medicines every day, knowledge of this should be further expanded. The deceases of the patients with the functioning graft who were diagnosed with the cardiovascular system diseases, constitute 50% of all renal transplant recipients. Immunosuppressive treatment leads to many pathological alterations within the organs and tissues and additionally they undoubtedly affect the activity of MMPs in the wall of the vessels.

## Introduction

Nowadays, organ transplantations are being carried out increasingly often. Following the transplantation procedure, patients take immunosuppressive agents which prevent transplant organ rejection. Contemporary immunosuppressive treatment constitutes of 4 groups of medications, such as: calcineurin inhibitors (CNIs) including cyclosporine A (CsA) and tacrolimus (TAC); inhibitors of the inosine monophosphate dehydrogenase (IMDH inhibitors) - the only agent from this group currently used in transplantation is mycophenalate mofetil (MMF); mTOR inhibitors (mTORIs), consisting of everolimus and glucocorticosteroids (GCS) 1. On one hand, immunosuppressive agents lower immunity, on the other hand, they display toxic impact on the organs 2-7. Furthermore, they can lead to pathological changes within the vessels 8, 9. The dysfunction of the endothelium is currently regarded as one of the dominant aspects of vessel change development. Recently, increase of interest in the influence of immunosuppressive treatment on the disorder of cardiovascular system is clearly observed, with the particular focus on the role of vessel endopeptidases. Chronic graft dysfunction (with both immunological and non-immunological background) accounts for 50% of the organ rejections, whereas the deceases of the patients with the functioning graft who were diagnosed with the cardiovascular system diseases, infections or tumors constitute the other half 10.

The cascade of the ongoing processes is mediated by means of the metalloproteinases (MMPs). Metalloproteinases have significant role in the transformation and functioning of the extracellular matrix of numerous tissues and organism structures. The current study focuses on the role of MMPs in the pathogenesis of the vascular damage and at the same time it offers the review referring to the influence of the immunosuppressive treatment on this interdependence.

## Metalloproteinases

Extracellular matrix (ECM) is a multi-component solid structure which fills the space between the cells. It consists of the ground substance and fibers. The ground substance is an amorphous substance which binds significant amounts of water. Apart from the glycosaminoglycans and proteoglycans, it comprises glycoproteins suspended in the polysaccharide solution, which gives the function of ideal framework for the tissues as well as safe environment for the maturing cells. Extracellular matrix is also a very active structure, subject to constant changes and transformations, simultaneously controlling and modulating the signals reaching the cells 11.

Dynamically transforming ECM structure plays significant role both in proper functioning of the tissues as well as in the pathomechanism of various organ ailments, including pathogenesis of the cardiovascular system diseases. The fundamental role in the pathological transformation of extracellular components, in the vascular remodeling as well as in the migration and proliferation of cells, is attributed to the malfunctions of metalloproteinases (MMP) and metalloproteinases inhibitors (TIMPs) 12-14.

Matrix metalloproteinases (MMPs) are structurally similar to endopeptidases whose activity impacts the structure of ECM proteins and connective tissue. Their proteolytic activity is not limited to the transformation of extracellular sphere of tissues. Dissecting numerous proteins, they contribute to the degradation of receptors on the cellular surface creating peptide fragments which bind numerous mediators. Through the protein hydrolysis they modulate activity and concentration of growth factors and cytokines. The purpose of their activity is to create the space which enables migration of cells and proper regulation of matrix-cell and cell-cell interactions as well as the arrangement of matrix structure 15-18. It plays significant role in many physiological processes, including embryogenesis, angiogenesis, remodeling of vessels. Keeping proper balance between the processes of degradation and synthesis of extracellular matrix components constitutes the basic requirement for maintaining tissue balance. Uncontrolled increase of MMP expression and activity is the characteristic component for the ongoing alterations with the pathological background 16, 19-21.

MMPs classification is primarily based on their activity, various substrate specifics enabled the classification of various endopeptidases to one of the six distinguished groups: collagenase - MMP-1, MMP-8, MMP-13; gelatinase - MMP-2, MMP-9; stromielisin - MMP-3, MMP-10, MMP-11; matrilisin - MMP-7, MMP-26; membrane type metalloproteinases - MMP-14, MMP-15, MMP-16, MMP-17, MMP-24, MMP-25; non-classified metalloproteinases - MMP-12, MMP-19, MMP-20, MMP-21, MMP-23 16, 22-24. Classification is also based on the difference in the protein quaternary structure, however general structure pattern is homologous for all metalloproteinases. Configuration of these molecules consists of three domains: n-terminal domain built of signaling peptides (separated after translation), propeptid, catalytic domain with the active spot and C-terminal domain 24.

In majority, MMPs are released to the extracellular space in the form of the inactive proenzymes- zymogens. N-terminal domain is in charge of maintaining enzyme in this form. Cysteine, which is included in the propeptid, binds with zinc atom in the active site of the catalytic domain by thiol groups. The whole entity is stabilized by three molecules of histidine in the active site. The first stage of the metalloproteinase activation involves cysteine separation from N-terminal domain and ultimate separation of propeptide from MMP. The final hemopexin domain is located on the C-terminal end. It also takes part in the endopeptidase mobilization, and due to the inhibitors adding capacity, it impacts inhibition of the process 15, 16, 25-28.

## Regulation of MMPs expression and activity

Inhibition of active enzymes is carried out by means of nonspecific (α-2 macroglobulin and α1-antitrypsin) and specific inhibitors. Four endogenous metalloproteinases inhibitors (TIMPs) - TIMP-1, TIMP-2, TIMP-3, TIMP-4 belong to the latter group 29. They display various affinity in reference to MMPs. Similarly to endopeptidases, these proteins consist of domains. Due to C-end domain they connect with metalloproteinases in 1:1 ratio. This inhibition form is a reversible process 26, 30-33. Metalloproteinases activation may be also controlled in the gene transcription stage, zymogenes release and proenzymes activation. This stimulation is activated under the influence of various factors activity, i.e. cytokines (IL-1, IL-6), growth factors (EGF, FGF, VEGF, PDGF, HGF), tumor necrosis factors (TNF-α, TNF-β), antigens (CD40), free radicals, plasmines, trombines, urokinase, nitrogen oxide 34-38.

**Figure 1:**
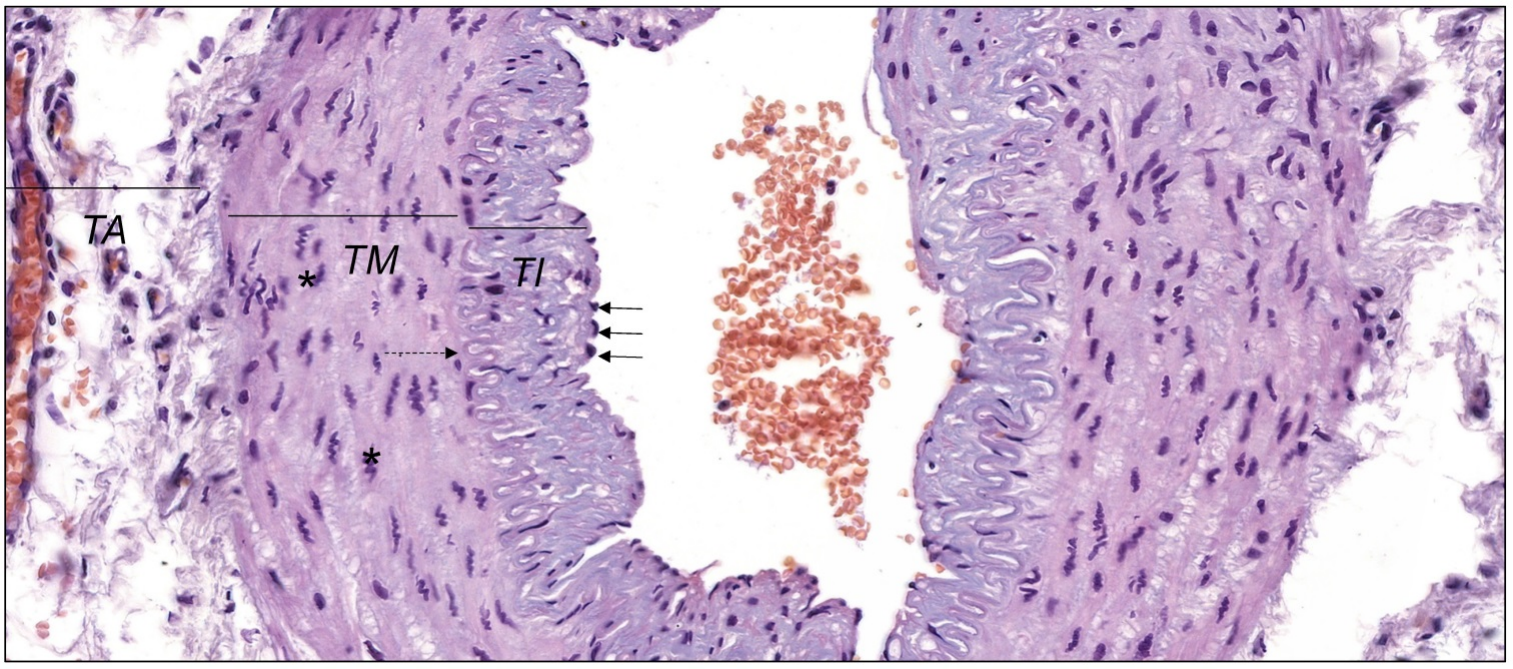
The wall of muscular artery presenting *tunica intima* (*TI*) lined with endothelium with nuclei of endothelial cells (black arrows), *tunica media* (*TM*) with nuclei of vascular smooth muscle cells (* ) and *tunica adventitia* (*TA*). Between *TI* and *TM internal elastic lamina* is visible (dashed arrow). Hematoxylin and eosin staining (objective magnification, ×40). Original photography by Aleksandra Wilk, Department of Histology and Embryology, Pomeranian Medical University in Szczecin, Poland.

In the physiological conditions, endothelial cells and smooth muscle cells control release of MMPs and TIMPs on the constant level. It enables to keep the balance between degradation and synthesis of the blood vessel wall elements. The histological structure of the wall of muscular artery was presented in Figure 1. Each type of endothelium damage results in the activation of the inflammatory cascade and the adhesion of the monocytes and macrophages to the vessel wall results in the violation of stability and the uncontrolled increase of MMPs activity 31, 39, 40. Additionally, the activity of endopeptidases is strengthened by constant growth of the proinflammatory cytokines, tumor necrosis factors or oxidized lipoproteins with low density. The activity of metalloproteinases itself is based on setting the path for the inflammatory cells, destruction of the extracellular matrix proteins as well as stimulation and proliferation of smooth muscles cells 39, 41.

It has been confirmed that gelatinases (MMP-2, MMP-9) influence the development of atherosclerotic plaque and, moreover, they influence the origination of aneurysms. Expression of the remaining peptidases varies according to the kind and structure of the vascular lesions. Nevertheless, infiltration from the inflammatory cells and increased expression of the relevant MMPs constitute progression markers of the atherosclerotic plaque and the indicators of its instability 39, 41-43. This interdependence is the reason of current increase of interest in metalloproteinases as the prognostic factors in course of cardio-vascular diseases.

## Clinical significance of metalloproteinases in the vascular diseases

Distinct increase of MMP-1, -2, -3, -8, -9, -10, -11, -12, -13, -14, -16 activity can be diagnosed within atherosclerotic changes 42, 44. According to the aforementioned, the dominance of individual peptidases differs according to the structure of the atherosclerotic plaque. In case of the fibrous changes, MMP-1, MMP-3 and MP-9 dominate but the changes rich in lipids are characterized by increased levels of MMP-1, MMP-8, MMP-12, MMP-12, MMP-16 42, 44. Clinical significance of the metalloproteinases in the vascular diseases aims to show the correlation between excessive activity of individual peptidases in the blood serum and prove their connection with the patient's disease condition.

The table below presents the summary of the research carried out so far on the potential increase of MMPs in the selected vascular diseases (Table 1).

## Immunosuppressive agents influence on MMPs activity in the blood vessels

This part of review describes the influence of immunosuppressive drugs with the division on calcineurin inhibitors and mTORs on MMPs activity in the wall of the blood vessels. Additionally, the summary of is presented in Table 2.

## Calcineurin inhibitors

Calcineurin is the protein that exhibits serine-threonine phosphatase activity. The most significant function of this enzyme is the transfer of signals in the path leading to the activation of the T-lymphocytes 56. In order to show its role in the functioning of the immunological system, it is necessary to explain shortly the structure and activity of this protein. Calcineurin is composed of two major sub-units: calcineurin A with the catalytic function to bind calmodulin, and calcineurin B- regulatory entity connecting calcium. The recognition of antigen by the T-cell receptors, through the activity of phospholipase C and inositol triphosphate, leads to the increase of cytoplasmatic concentration of calcium in the cell 6. Calcineurin-calmodulin-calcium complex originates, which activates calcineurin. Subsequently, calcineurin influences expression of relevant genes by dephosphorylation of nuclear factors of the activated T-cells (NFATc) 9, 57. Dephosphorylated forms of enzymes migrate from the cytoplasm to cell nucleus where they lead to, *inter alia*, increase of gene transcription for IL-2, IL-3, IL-4 and tumor necrosis factor (TNF-α). Created cytokines stimulate proliferation and differentiation of leukocytes. Calcineurin inhibitors act by stopping the described sequence. Due to the strong immunosuppressive activity they are a standard practice in the treatment process following the organ transplantation, which is highly effective in the prevention of the graft rejection. Moreover, they display numerous negative symptoms, inter alia, adverse effect on the cardio-vascular system 9, 58. Cyclosporin A (CsA) and Tacrolimus (Tac) belong to the calcineurin inhibitors group of medicaments.

Immunosuppressive activity of CsA is based on binding calcineurin with cyclophilin. It prevents dephosphorylation NFATc and thereby its translocation to nucleus and gene transcription to, inter alia, IL-2 6, 59. CsA also blocks signaling pathways activating T-lymphocytes, to a lesser extent it influences the inhibition of antibodies production and reduction of mobilization of macrophages 4. Apart from the influence on the immunological system, this medicament demonstrates a number of adverse effects, including acute and chronic nephrotoxicity and arterial hypertension. These complications result from the damage to the vessel 2, 5, 6, 59.

The researches carried out using human umbilical vein endothelium cells (HUVECs) demonstrated increase of MMPs activity in the cytoplasm. Isolated HUVECs cells were put on the platelets and incubated with cyclosporin. The results of this study may suggest adverse effect of CsA on the vessel structure by the violation of balance of the specific endopeptidases: activity increase of MMP-1, MMP-3, MMP-8, MMP-9, MMP-13 and activity decrease of MMP-2 60. Furthermore, research carried out on the rats arteries demonstrates more extensively the influence of CsA on the vessel's wall. The animals which underwent balloon angioplasty of the common carotid artery were given cyclosporine. Spot thickening of the endothelium connected with the increased depositing of ECM components was observed among the animals treated with CsA, and in consequence vessel lumen was narrowed. MMP-2, MMP-9 and TIMP-1 expression was lowered in comparison to the control group, however the ratio MMP-2:TIMP-1 and MMP-9:TIMP-1 was increased in both cases 61. Moreover, myocardium is also subjected to the adverse influence of CsA. In the cardiac muscle tissue of rats which underwent the treatment several degenerative changes accompanied with fibrosis were observed. MMP-2 level increase was accompanied with the increase of vessel endothelium growth factor- VEGF. The authors suggest that both proteins are the response against excessive amount of collagen and ischemic episodes resulting from CsA toxic activity. The response of the myocardium to the excessive growth of ECM is the increased enzymatic response 62.

Regarding CsA, recent studies report positive influence of the mesenchymal stem cells (MSCs) on the improvement of the activity of the kidney subjected to the ischemic-reperfusion operation and subsequent therapy using CsA. Ischemia-reperfusion model of kidney was obtained in rats subjected to unilateral nephrectomy. Then, for the period of 28 days, the animals were given CsA. MSCs was injected directly to the kidney for 7 days after ischemic-reperfusion operation. Interestingly, the effect of giving mesenchymal cells was connected with the decrease of infrestitial fibrosis, improved activity of kidneys and lower activity of MMP-2 in comparison with organs which underwent immunosuppressive treatment only. In summary, applying MSC may be connected with the protective influence on the organ subjected to stressful factors such as ischemia or toxic effect of CsA 63.

Tacrolimus is the second important representative from the group of calcineurin inhibitors. Contrary to CsA connected with cyclophilin, this drug interacts with the protein binding tacrolimus- FKBP. Immunosuppressive mechanism remains similar, it prevents dephosphorylation of the transcriptive factors and, in consequence, cytokine expression and cascade stimulating proliferation and T-lymphocyte activity is inhibited 59, 64.

Comparison of the influence of immunosuppressants on the expansion of intima membrane among rats subjected to the balloon angioplasty of the carotid artery, tacrolimus (in contrast to CsA) did not result in the thickening of the intima membrane of the vessel. At the same time, it inhibited expression of MMP-2, MMp-9 and TIMP-1. The violation of balance of metalloproteinases release reflected in the increased depositing of ECM components within the vessel wall 61. In the study presenting diverse influence of cyclosporin and tacrolimus on the gene expression associated with the fibrosis in the isolated renal glomeruli of the kidney recipients higher profibrotic activity of CsA than Tac was also observed. Extracellular matrix was constantly remodeled. The balance between synthesis and degradation of its components may have been maintained by the action of MMPs. Increase of TIMP release supports excessive expansion of ECM. Increased mRNA level for collagen III and TIMP-1 in comparison CsA was observed among the patients treated with Tac, however no disparity was reported in the expression of MMP-2 and TIMP-2 between the tested groups 65. Additionally, expression level of profibrotic genes among the patients with cyclosporine- or tacrolimus- induced nephrotoxicity was higher among the patients treated with tacrolimus. It was probably associated with higher expression of MMP-2, TIMP-2 and collagen in the transplanted organ 66.

Fibrosis remains the serious problem in aspect of immunosuppressive treatment. The therapy with the application of pirfenidone- antifibrotic compound probably inhibits the processes leading to fibrosis. In the study which examines the inhibitory effect of pirfenidone on the expression of profibrotic genes in the nephrotoxicity induced by tacrolimus model, the application of sole inhibitor was compared with the therapy based on pirfenidone. Decrease of TIMP-1 expression and lack of effect on the expression of collagen, MMP-2 and MMP-9 in the rats kidneys were reported in monotherapy, whereas, two drugs resulted in the higher decrease of TIMP-1 and collagen expression 67.

Another widely applied group of immunosuppressants are mTOR pathway inhibitors. mTOR protein is threonine-serine kinase, the major task of which is maintaining control on proliferation, growth and motion of cells. Rapamycin inhibits mTOR pathway by interacting with FKPB-12 receptor 3. It prevents the translation of proteins involved in the cellular cycle progression: T- and B-lymphocytes remain in G1 phase 3, 4. Antiproliferation activity of rapamycin is also applied in cardiology. Inhibition of division and migration of smooth myocytes positively affects remote test results among the patients who underwent angioplasty of the coronary arteries. Stents coated with rapamycin prevent restenosis much more effectively than the classic ones 4.

In the study of Waller et al. 61 on the influence of immunosuppressants on the rats arteries, rapamycin turned out to exhibit the lowest level of toxicity. Despite similar expression level of MMP-2, MMP-9, TIMP-1 and ECM accumulation, it was the only drug which did not cause the thickening of the *tunica intima* of the wall of aorta. The depositing of the ECM was less marked in case of the application of rapamycinwall in contrast to the vessel walls among the animals treated with CsA 61. Additionally, rapamycin displays inhibitory effect on cell migration activity, what was described by Gao et al. 68. The results of the aforementioned study suggest that rapamycin inhibits cell migration and ECM degradation by inhibiting endothelial-to-mesenchymal transition and the endothelial cell secretion of MMP-2 and MMP-9. These processes may be related to possible mechanisms for the inhibition of angiogenesis by rapamycin 68.

The inhibitory impact of rapamycin on the reconstruction of vessels and expression of genes associated with the fibrosis in the model of transplantation of the aortas of rats was also observed 69. The ascending segment of the thoracic aorta taken from the f344 rats was transplanted to the Lewis rats for the anastomosis with the abdominal aorta to make a loop. After the surgery, the animals were treated with rapamycin in two doses 0.25 mg/kg/daily and 0.5 mg/kg/daily. The application of higher dose did not cause hyperplasia of the intima membrane, it inhibited depositing of the ECM and did not display expansive restructuring of the vessels in comparison to the control groups. The decrease of the MMP-2, MMP-9 collagen III and TMP-1 expression was noticed. Lower effect of the drug effect on the restructuring of the vessels was observed among the animals treated with lower dose, which resulted in bigger inflammatory lesion and increased expression of MMP-9. It suggests that rapamycin may have profound influence on the inhibition of remodeling of the vessels which have been damaged. It is of great importance then to administer properly selected dosage which will stop the accumulation of the inflammatory process and thus remodeling 69.

Furthermore, in the studies on aneurysm of aorta, inhibitory effect of rapamycin on the progression of change was observed. The rats which were given rapamycin presented 40% decrease of aneurism progression in comparison to the control group. Interestingly, much lower MMP-9 activity among the rats subjected to mTORs was reported 70.

## Conclusions

Due to the fact that the properties of immunosuppressive drugs still remain unclear and transplant recipients need to use these medicines every day, knowledge of this should be further expanded. The deceases of the patients with the functioning graft who were diagnosed with the cardiovascular system diseases, constitute 50% of all renal transplant recipients. Immunosuppressive treatment leads to many pathological alterations within the organs and tissues and additionally they undoubtedly affect the activity of MMPs in the wall of the vessels.

## Figures and Tables

**Table 1:** Individual MMP activity in the disease entities.

Disease entity	Increase of enzyme	Reference
Coronary heart disease	MMP-1, MMP-2, MMP-8, MMP-9	45-47
Unstable coronary heart disease	MMP-1, MMP-2, MMP-9, MMP-3	45, 46, 48, 49
Arterial hypertension	MMP-1, MMP-9, TIMP-1	50-52
Aorta aneurysm	MMP-3, MMP-9	46, 53, 54
Atherosclerosis of the peripheral vessels	MMP-2, MMP-9	44, 55

**Table 2:** Influence of immunosuppressants on MMPs activity

Drug	Metalloproteinases activities	Examined material	Comments	References	Country
Calcineurin Inhibitors					
Cyclosporine	Increased activity of MMP-1, MMP-3, MMP-8, MMP-9 and MMP-13, decreased activity of MMP2	Endothelial cells of human umbilical vein	Tests carried out on the umbilical vein endothelial cells	60	Korea
	Inhibition of activity of MMP-2, MMP-9, TIMP-1,	Carotid arteries of rats	Local thickening of the endothelium. Increased depositing of ECM in the vascular endothelium	61	UK
	Increase of MMP2 activity and expression following renal ischemia- reperfusion and treatment with cyclosporine	Kidneys of rats	Progression of lumen of the vessel narrowing	63	France
	MMP2 level increase, constant levels of MMP-1, MMP-9	Myocardium of rats	Degenerative changes with the fibrosis of myocardium and increase of MMP2 and VEGF.	62	Italy
Tacrolimus	Inhibition of activity of MMP-2, MMP-9, TIMP-1,	Carotid arteries of rats	No effect on the local thickening of the endothelium. Increased depositing of ECM in the inner layer of the vessel.	61	UK
	Expression of Informational RNA (mRNA) for collage III and TIMP-1was significantly lower among the patients who were given tacrolimus than among the patients treated with cyclosporine.	Renal biopsies from the post-transplant patients	-	65	UK
	Intrarenal expression of MMP-2, TIMP-2 increased among the patients treated with tacrolimus in comparison to the patients treated with cyclosporine.	Renal biopsies	Renal biopsies from the patients with the histologically diagnosed CsA or Tac nephrotoxicity CsA or Tac and acute rejection.	66	USA
	Expression of MMP-2 and MMP-9 remained unchanged by tacrolimus, TIMP-1 expression was lowered.	Rats kidneys	Test results suggest that pirfenidone may additionally lower fibrosis potential of tacrolimus	67	UK
mTOR inhibitors					
Rapamycin	Inhibiting the activity of MMP-2, MMP-9, TIMP-1,	Rats carotid	Decrease of the endothelium local thickening. Increased depositing of ECM depositing in the inner layer of the vessel. Decreasing the progression of vessel narrowing.	61	UK
	Inhibiting the release of MMP-2, MMP-9.	The endothelium cells of the human umbilical vein	Tests carried out on the human pathway of the endothelium cells of the umbilical vein EA.hy926. inhibition of cellular migration and degradation of the extracellular matrix. Profibrotic activity.	68	China
	Inhibiting activity of MMP-9	Experimentally developed renal artery aneurysms in rats	Decreased progression of aneurism following rapamycin application	70	USA
	Inhibiting activity of MMP-2, MMP-9, TIMP-1	Allogenic transplantations of rats' thoracic aorta	-	69	UK
